# *Helicobacter pylori* Is Not Eradicated after Triple Therapy: A Nested PCR Based Study

**DOI:** 10.1155/2014/483136

**Published:** 2014-06-23

**Authors:** Saurabh Kumar Patel, Girish Narayan Mishra, Chandra Bhan Pratap, Ashok Kumar Jain, Gopal Nath

**Affiliations:** ^1^Department of Microbiology, Institute of Medical Sciences, Banaras Hindu University, Varanasi, Uttar Pradesh 221005, India; ^2^Department of Medicine, Institute of Medical Sciences, Banaras Hindu University, Varanasi, Uttar Pradesh 221005, India; ^3^Department of Gastroenterology, Institute of Medical Sciences, Banaras Hindu University, Varanasi, Uttar Pradesh 221005, India

## Abstract

Detection of *Helicobacter pylori* after triple therapy is usually carried out by either rapid urease test (RUT), urea breath test (UBT), histology, bacterial isolation, and single round PCR or serological tests. In this study, antral biopsy specimens from 25 patients were tested for *H. pylori* by RUT, culture, histology, and nested PCR in their antral biopsy specimens before and after treatment. Three genes, namely, heat shock protein (*hsp60*), phosphoglucosamine mutase (*ureC*), and flagellar export ATP synthase (*fliI*) of *H. pylori* were targeted. Of the 25 antral biopsy specimens, the RUT, culture, histology, and nested PCR positivity dropped from 81.8% to 12%, 31% to 0%, 100 to 84%, and 100% to 92%, respectively, before and after therapy. Further, *hsp60* specific amplicons from 23 out of 25 patients gave identical restriction pattern, while 6 *fliI* and 1 *ureC* specific amplicon produced different restriction pattern. Furthermore, variations in *fliI* gene sequences in *H. pylori* after treatment were also confirmed by sequencing and compared *in silico*. Nested PCR based detection of *H. pylori* is more sensitive method to detect *H. pylori* after therapy than culture, RUT, and histology. Further, this study suggests that *H. pylori* is not eradicated completely after triple therapy.

## 1. Introduction

Association of* Helicobacter pylori* (*H. pylori*) with acid peptic diseases including duodenal ulcer is well established [1]. Further,* H. pylori* has been designated as class I carcinogen by WHO [2]. Although, prevalence of* H. pylori* ranges between 20 and 80% in different geographical areas depending on different socioeconomic factors, eradication has been advised only in symptomatic cases [3–6]. Triple therapy constituting omeprazole, amoxicillin, and clarithromycin has been found to be quite effective to eliminate* H. pylori* from stomach [7, 8]. However, occasional recurrences have been reported with isolation of strain similar to that of pretreatment [9–13]. It is not clear whether the infection persisted after triple therapy or reinfection occurred from the other niches, for example, oral cavity, or acquired from other close family members. For primary diagnosis and posttherapy evaluation of* H. pylori*, most of the studies have used rapid urease test (RUT)/CLO test, histology, C^13/14^-urea breath test (UBT), and culture and stool antigen detection test. Because of poor sensitivity of isolation of* H. pylori*, histology and UBT are usually considered as gold standard for the assessment of eradication therapy. But these tests are found sensitive enough only when density of* H. pylori* remains high. Contrary to this, PCR based technique can detect even a few bacteria. There are scant reports using single round PCR based detection of* H. pylori* after therapy. It has already been established that the sensitivity of nested PCR based detection is very high in comparison to single round [14]. Interestingly, there is no report till date by using nested PCR based detection of the bacterium after eradication therapy in* H. pylori* associated diseases. Therefore, present study was planned to see whether,* H. pylori* is really eradicated by using nested PCR protocol and if not, whether the persistent strains are the same or different.

## 2. Methods

### 2.1. Patient Selection

This study was conducted at Sir Sunder Lal Hospital, Banaras Hindu University, Varanasi, during June, 2009 to March, 2010. Ethical committee clearance was obtained before commencement of the study and well informed written consent was obtained from each of the participants. The patients who had severe acid peptic diseases on endoscopy with positive test for* H. pylori* were given clarithromycin 500 mg, amoxicillin 1 gm, and pantoprazole 40 mg; all twice daily for 14 days. Patients were initially asked to visit again for follow up at 4 weeks after the completion of anti-*H. pylori* therapy. A total of 93 patients (63 male and 30 female; mean age 42.4 y; age range 20 to 85 y) were enrolled in the present study. Biopsies were collected in the endoscopy units of the Department of Gastroenterology. Patients taking proton pump inhibitors and/or antibiotics having bleeding ulcers or an acute hemorrhage from other sites in the upper gastrointestinal tract and patients with stomach surgery were excluded. The endoscope was mechanically washed and then disinfected using activated 2% glutaraldehyde. The 4-5 biopsy specimens were collected from each patient of the 93 enrolled patients. A total of 25 patients who had antral gastritis (*n* = 12) and peptic ulcer (*n* = 13) diagnosed previously could be followed up and upper gastrointestinal endoscopy was performed on both the occasions, that is, pre- and posttherapy, and biopsy samples were collected from gastric antrum. Since 68 patients did not report for the follow up after treatment, they were excluded and only 25 patient's antral biopsies could be analyzed in this study.

### 2.2. Rapid Urease Test (RUT)

For RUT, biopsy was inoculated into 1 mL of 10% urea dissolved in deionized water (pH 6.8), to which two drops of 1% phenol red solution were added and incubated at 37°C for 24 h. A positive result was recorded when the color changed from yellow to pink within 30 min [15].

### 2.3. Histology

Antral biopsy specimens, collected during pre- and posttreatment were fixed in 10% buffered formalin, embedded in paraffin. Paraffin sections were stained with hematoxylin and eosin to examine the presence/absence of curved rod shaped* H. pylori* on the mucosal surface.

### 2.4. Culture of* H. pylori* from Gastric Biopsy Specimens

The biopsy piece was homogenized into phosphate buffer saline (PBS) in an all glass disposable homogenizer. This tissue homogenate was plated onto the media containing brain heart infusion (BHI) agar (Difco, Becton Dickinson, Sparks, MD, USA), supplemented with 7% sheep blood, 0.4% IsoVitaleX, and Skirrow selective supplement (vancomycin 10 *μ*g/mL; polymixin B sulfate 2.5 IU/mL; trimethoprim lactate 5 *μ*g/mL) (Difco, Becton Dickinson, Sparks, MD, USA). Plates were incubated at 37°C in an atmosphere of 5% O_2_, 10% CO_2_, and 85% N_2_ for 3–7 days. Plates were opened after 72 h and every 24 h afterwards, if no growth was obtained. Plates were discarded only after 7 days of incubation. Organisms were identified as* H. pylori *based on typical colony morphology, Gram staining, and positive oxidase, catalase, and rapid urease tests [15].

### 2.5. Preparation of Genomic DNA for PCR Assay

Genomic DNA from tissue homogenate was extracted using a standard proteinase K and phenol-chloroform method [16]. One set of double distilled water was included in each batch of DNA extraction to check cross-contamination of DNA during DNA extraction.

### 2.6. PCR Amplification

PCR was carried out in a 25 *μ*L volume using 10 ng of DNA, 1 U of Taq polymerase (Bangalore Genie, India), and 10 pmol of each primer (SBS Genetech), 0.25 mM (each) deoxynucleotide triphosphate, and 1.5 mM MgCl_2_ in standard PCR buffer. For the internal amplification, the PCR product from the primary cycle was diluted 1/50 and 1 *μ*L was used as the template in the nested PCR [15]. All the amplifications were carried out in a thermal cycler (Biometra, Goettingen, Germany). Details of primers and their protocol are given in Table 1. Amplification of all the three conserved genes were carried out by nested protocol. Universal eubacterial primers were used for all the samples to exclude PCR inhibition. DNA from* H. pylori* reference BHUHPSKP3 (KC525436) and a tube containing water in place of DNA were assayed in each PCR run as positive and negative controls, respectively. The PCR products were analyzed by electrophoresis on 1.4% agarose gels (Bangalore Genie, India) containing 0.5 *μ*g of ethidium bromide per mL. The gel was run at 70 V with TBE (Tris Boric acid EDTA) buffer and was examined by transilluminator and photographed.

### 2.7. PCR Based Restriction Enzyme Analysis (PCR-REA)

After amplification, the PCR products [*hsp60, ureC* or* glmM,* and* fliI* gene] were precipitated with 2.5 volumes of ethanol. The pellets were washed twice with 75% ethanol and dissolved in Tris-EDTA buffer (pH 8.0). A 10 *μ*L precipitated amplified DNA was then digested with the 10 U of restriction enzyme in appropriate buffered solution recommended by the manufacturer (Genie, Bangalore, India) and incubated for 3 h at 37°C.* Hin*d III restriction enzyme was used for* hsp*60 and* ureC,* and* Mnl *I was used for* fliI* gene. The digested DNA fragments were analyzed by electrophoresis on 2% agarose gels (Genie, Bangalore, India) containing 0.5 *μ*g of ethidium bromide per mL. The gel was run at 70 V with TBE (Tris Boric acid EDTA) buffer for 3 h and was examined by transilluminator and photographed. The sizes of digested DNA fragments were estimated from migration distances of a 100-bp DNA ladder molecular weight standard (Genie, Bangalore, India) and compared with* in silico* restriction digestion specified with concerned restriction enzyme.

### 2.8. Sequencing

The amplified* fliI* gene segment, which had different restriction pattern from previous strain were purified from salts and primers using HiPura silica kit for DNA isolation (HiMedia). A total of 6 (5 mutated and 1 wild type) purified amplicons generated targeting* fliI* were outsourced for partial sequencing to Genei, Bangalore, India. Sequences were analyzed using BLASTN (http://www.ncbi.nlm.nih.gov/BLAST/) to verify mutations/changes in the sequences of* fliI *gene specific for* H. pylori*.

## 3. Results

### 3.1. Bacteriological Study

Of the 25 antral biopsy specimens collected from patients, 81.8% (18/25) were found positive by RUT and 31% (7/25) by culture for* H. pylori, *before triple therapy. Four weeks after anti-*H. pylori* triple therapy, 3 (12%) patients (2 PUD and 1 gastritis) were positive by RUT and none of them were positive for* H. pylori* isolation after triple therapy.

### 3.2. Histology

Of the 25 patients with gastroduodenal diseases that completed eradication treatment, 16% (4/25) were still* H. pylori* positive by histology.

### 3.3. Nested PCR

**Figure 1:**
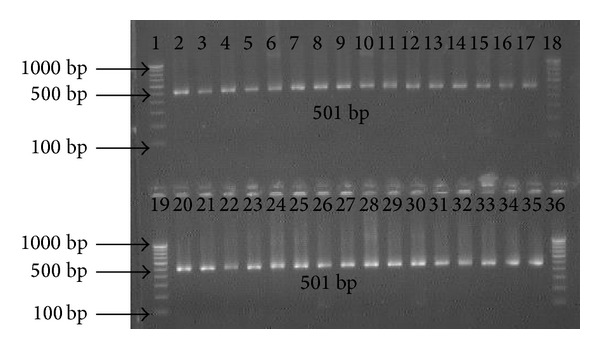
Amplification of partial 501 bp* hsp60* gene with specific nested primer for* H. pylori* in antral biopsies. Lanes 1, 18, 19, and 36: molecular marker (100 bp); lanes 2 and 20: positive control; lanes 3 to 17: gDNA from antral biopsies before treatment; and lanes 21 to 35: gDNA from antral biopsies after treatment.

**Figure 2:**
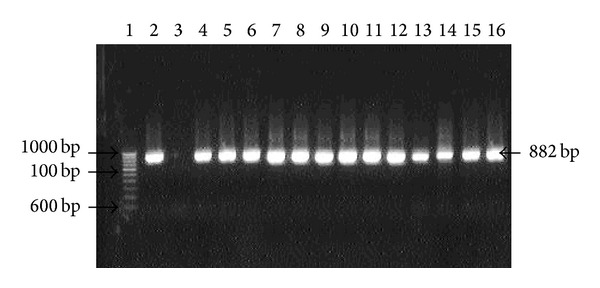
Amplification of* ureC* (*glmM*) gene with internal nested primer specific for* H. pylori* in antral biopsies. Lane 1: molecular marker (100 bp); lane 2: positive control; lane 3: negative control; lanes 4 to 16: gDNA from antral biopsies collected after treatment.

**Figure 3:**
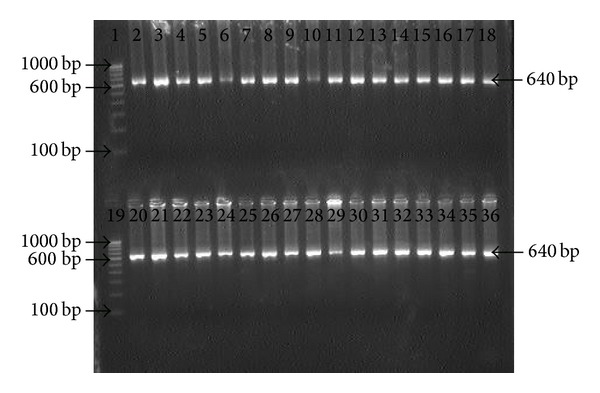
Electrophotograph showing amplification of* fliI* gene with specific internal primer for* H. pylori* in antral biopsies. Lanes 1 and 19: molecular marker (100 bp); lanes 2 and 20: positive control; Lanes 3 to 18: gDNA from antral biopsies before treatment; and Lanes 21 to 35: gDNA from antral biopsies after treatment.

Genomic DNA extracted from biopsy specimens were subjected to amplification by primers specific for* hsp60, ureC,* and* fliI* genes of* H. pylori. *All the 25 patients were positive for the 501 bp, 840 bp, and 640 bp of amplicon for* hsp60, ureC,* and* fliI* genes, respectively, before therapy. However, 92% (23/25) antral biopsies were positive for* H. pylori *gDNA by nested PCR after 4 weeks, while 2 patients were found negative (Figures 1, 2, and 3).

### 3.4. PCR-REA

**Figure 4:**
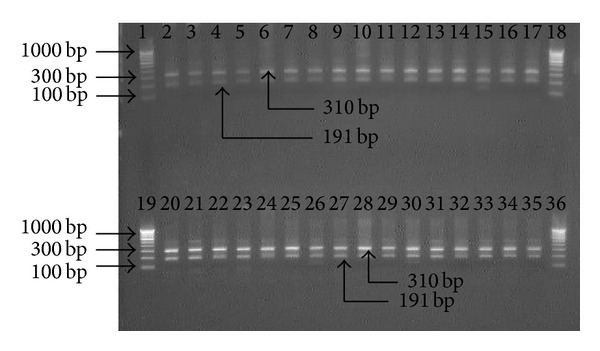
Electrophotograph of restriction digestion of* hsp*60 gene of* H. pylori* strains with* Hin*d III, restricted 501 bp* hsp60* gene amplicon into two fragments (310 and 191 bp). Lanes 1, 18, 19, and 36: 100 bp molecular marker; lanes 2 to 17: restriction pattern of PCR product specific to* hsp60* before treatment; lanes 20–35: restriction pattern of amplicons specific to* hsp60* gene after treatment.

The amplified PCR products* of hsp60* gene were digested by* Hin*d III enzyme. All nested amplicons of 501 bp were restricted into two fragments of 310 and 191 bp by the* Hin*d III restriction enzyme. There was no difference in restriction pattern of amplified partial* hsp60* gene of strains of* H. pylori* before and after treatment (Table 2; Figure 4).

**Figure 5:**
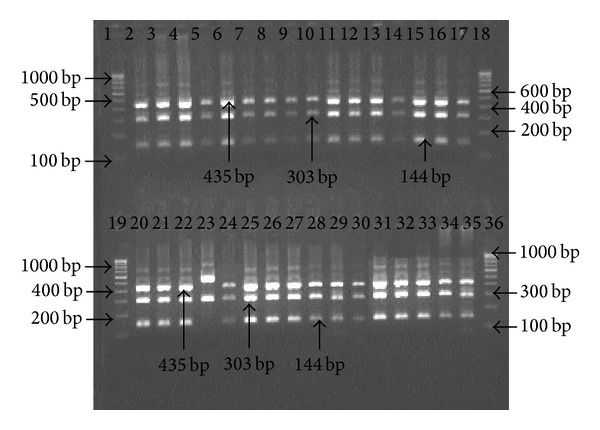
Electrophotograph of restriction analysis of* ureC *(*glmM*) gene of* H. pylori* strains with* Hin*d III, restricted 882 bp* ureC* gene amplicon into 3 fragments (435, 303, and 144 bp). Lanes 1, 18, 19, and 36: 100 bp molecular marker; lanes 2 to 17: restriction pattern of PCR product specific to* ureC* before treatment; lanes 20–35: restriction pattern of amplicons specific to* ureC* gene after treatment.

The amplified PCR products of* ureC* gene were restricted by* Hin*d III enzyme. All amplified PCR products were restricted into 3 segments of 435, 303, and 144 bp. Strains amplified from all the patients were same except one patient with antral gastritis. The amplicon from this tissue restricted only into fragments of 303 and 579 bp (Table 2; Figure 5).

**Figure 6:**
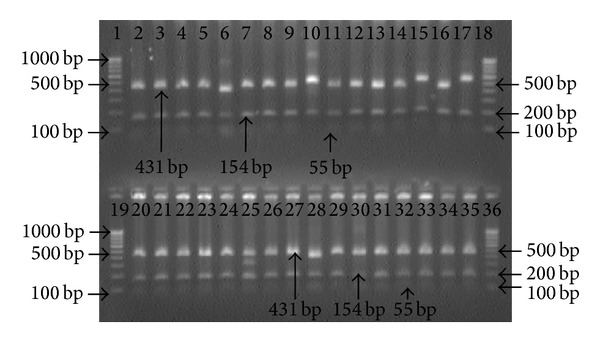
Electrophotograph of restriction digestion of* fliI* gene of* H. pylori* strains with* Mnl *I, restricted 640 bp* fliI* gene amplicon into three fragments (431, 154, and 55 bp). Lanes 1, 18, 19, and 36: 100 bp molecular marker; lanes 2 to 17: restriction pattern of PCR product specific to* fliI* after treatment; lanes 6, 10, 15, and 17 exhibited different band pattern comparison to their previous strains; and lanes 20–35: restriction pattern of amplicons specific to* fliI* gene before treatment.

The amplified PCR products of* fliI* gene were digested using* Mnl* I enzyme. Digestions of PCR product with* Mnl* I resulted into 3 fragments of 431, 154, and 55 bp similar to* in silico*. The DNA amplified from the 17 patients were identical, while 6 patients exhibited different restricted pattern than what were obtained before therapy (4 with peptic ulcer and 2 had antral gastritis). Restriction pattern of amplified* fliI* gene of* H. pylori* from 6 patients exhibited four different types. Three patients were type A, while remaining 3 strains belonged to each of the B1, B2 and C types. Type A strain showed two fragments of 486 and 154 bp. Type B was fragmented into three fragments 386, 154, and 55 bp, but on the ground of sequencing type B was further subdivided into two subgroups B1 and B2. Subtype B1 was restricted into 386, 154, 55, and 45 bp and subtype B2 was digested into 386, 134, 55, 45, and 22 bp. However, the smaller fragments could not be visualized on 1.8% agarose gel. Type C amplicon could be digested into 3 fragments, that is, 431, 300, and 154 bp (Table 2; Figure 6). However, this type could not be sequenced.

### 3.5. DNA Sequence of* fliI* Gene before and after Treatment and* In Silico *Restriction

**Figure 7:**
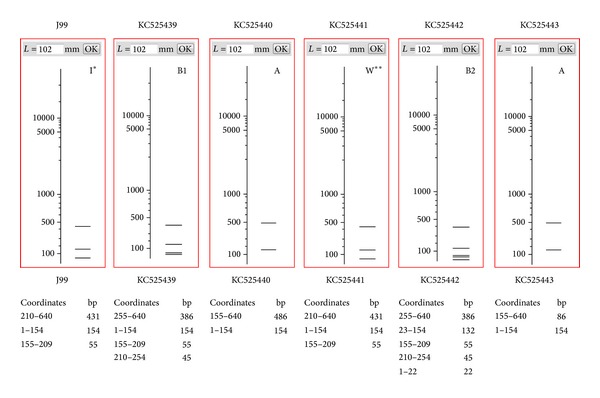
*In silico* restriction pattern of* fliI* gene (640 bp) specific to* H. pylori* digested with* Mnl* I. The virtual gel was generated by interpolating experimental data using NEB cutter V2.0. KC525441 (GenBank accession number) represents* in silico* restriction pattern like J99; KC525439, KC525440, KC525442, and KC525443 produced varying restriction pattern. I*: restriction pattern of J99; W**: wild type restriction pattern; A, B1, and B2: mutated/changed restriction pattern of* Mnl *I.

DNA sequences of* fliI* gene have been submitted to NCBI gene data Bank (GenBank accession number KC525439, KC525440, KC525441, KC525442, and KC525443). Comparison of the nucleotide sequences with the NCBI database showed 99% similarity with* H. pylori* flagellum-specific ATP synthase (*fliI*). The partial nucleotide sequence of* fliI* of 5 strains were flanked with nucleotide sequences from J99 as reference up to 640 bp similar to internal amplicon with assurance that no additional site could be generated during flanking. All the five sequenced nucleotides were restricted by* Mnl *I* in silico* (Figure 7).* In silico* restriction pattern was similar to experimental observation.

## 4. Discussion

The positivity for* H. pylori *in 23 of 25 patients (92%) who came for follow up after 4 weeks of completion of anti-*H. pylori* therapy is really surprising in the light of previous reports, where eradication rates ranged between 70 and 100%. This remarkable difference may be explained on the basis of relatively poor sensitivity of* H. pylori* detection by serological, UBT, fecal antigen, RUT, histopathology, culture, and single round PCR methods than by nested PCR. Anti-*H. pylori* antibody based method could demonstrate eradication rate of 85%, but it should be taken in the light of the fact that antibody fall may take time and also presence of* H. pylori* in body sites other than stomach cannot be excluded. UBT has been found to show eradication rates ranging between 75 and 100%. However, for positivity by UBT, urease producing bacterial density in stomach should be sufficient enough which is naturally reduced significantly by anti-*H. pylori* regimen. The same logic of low* H. pylori* density in stomach very well explains the quite high eradication rate assessed by fecal antigen detection, RUT, histology, single round PCR, and bacterial isolation methods. Further RUT, UBT and bacterial isolation primarily depends on viable and metabolically active form of* H. pylori*. But this is already established that the antimicrobial therapy not only causes reduction in bacterial load but also transforms active spiral bacteria to coccoid (viable but not culturable: VBNC) form [17, 18]. Further, single round PCR may give positive amplification only when more than 70 bacterial cells are present in a given biopsy sample [19], while nested PCR is capable of detecting the bacterium as low as 3 cells only [14]. In the present study, we targeted 3 genes (*hsp60, ureC,* and* fliI*) to rule out possibility of PCR contamination and all the targets gave specific amplification in each antral biopsies collected from 23 of the 25 patients. Further, we have taken full precaution to avoid cross-contamination through endoscopes by proper sterilization and performing PCR in 3 completely separated rooms. These observations suggest that extremely sensitive methods of* H. pylori* should be employed specifically for evaluation of therapeutic efficacy.

Further, we tried to see whether the* H. pylori* strains detected pre- and posttherapy are similar or they are the cases of reinfection by new strains. All amplicon originating from 3 different targets from each of 23 patients subjected to restriction analysis showed that pre- and poststrains were identical. However, one amplicon of* ureC* origin and 6 of* fliI* were found to give different banding pattern than the initial amplification experiment on antral biopsy in the same patient. Although possibility of rising mutations during therapy cannot be ruled out, majority of patients were found to harbor the same strain after 4 weeks posttherapy which suggests that complete* H. pylori* eradication has not occurred in these patients. Our observation goes in the same line as reported previously, where the authors have shown that infection of* H. pylori* persists after therapy [20–26]. However, reinfection by the bacterium having identical restriction pattern may occur a result of recolonisation of stomach originating from the oral cavity of the same patients or contacting infection from a family member harboring the same strain [27]. It may also be quite likely, however, that* H. pylori* may survive in the gastric pits where sufficient concentration of antibiotics may not be achieved or bacteria transformed to coccoid (VBNC) that makes antibiotic ineffective. In an animal model, Cellini et al. (1994) demonstrated that up to 3 months after inoculation viable but not culturable forms of* H. pylori* could still be detected in the mouse stomach [28]. A few studies [29, 30] were carried out to evaluate triple therapy comparing PCR with culture. However no report has included nested PCR. Interestingly, Hammar et al. (1992) described gastric biopsy samples that were* H. pylori* positive by PCR but negative by culture [19]. Similar findings of persistence of* H. pylori* antigens, detected both with single round PCR and enzyme immunoassay (EIA), in the stool of successfully eradicated patients have been reported [31].

Therefore, it may be proposed that* H. pylori* causes chronic infection and usually eradication does not occur by anti-*H. pylori* regimens. The symptomatic relief occurring in the patients might be due to overall reduction in the bacterial density which might have aggravated the problem. Further, the possibility of presence of other bacteria than* H. pylori* causing acid peptic disease which are taken care off by the same antimicrobial agents may be also considered.

In conclusion, conventional methods to detect* H. pylori* especially posttherapy could not detect the pathogens as can be done by nested PCR protocol. Therefore, nested PCR may be proposed as the gold standard. Moreover, RUT, UBT, and histopathology are unable to discriminate the reinfection or recrudescence, while PCR based method (restriction analysis or sequencing) has capability to indicate either of the two possibilities.

## Figures and Tables

**Table 1:** Primers used for detection of *H. pylori* before and after treatment targeting specific genes.

S. number	Targeted gene		Primer sequence (5′-3′)	Product size	PCR condition, (annealing temperature and cycles) MgCl_2_ conc.
1	Heat shock protein (*hsp60*)^14^, conserved	Primary	AAGGCATGCAAGATAGAGGCT CTTTTTTCTCTTTCATTTCCACTT	590 bp	94°C, 30 seconds, 56°C, 30 seconds; 72°C, 30 s (35 cycles) (60 mol/lit)
		Nested	TTGATAGAGGCTACCTCTCC TGTCATAATCGCTTGTCGTGC	501 bp	94°C, 30 seconds, 56°C, 30 seconds; 72°C, 30 s (35 cycles) (60 mol/lit)
2	Phosphoglucosamine mutase (*ureC/glmM*), conserved	Primary	TTGGGGGTATAATTCAAGGG TTAGTGAGCGCTCTAACTTCC	945 bp	94°C, 1 min, 59°C, 1 min; 72°C, 1 min (35 cycles) (60 mol/lit)
		Nested	GCAACAGAGCTTACCTGCTTG GATTCAAATAGGGCCTATGC	882 bp	94°C, 1 min, 59°C, 1 min; 72°C, 1 min (35 cycles) (60 mol/lit)
3	Flagellum-specific ATP synthase (*fliI*)∗, conserved	Primary	CCCGATGCGAATGAGCATTTC GCTTAACCCTTTAGGGCAAGTC	858 bp	94°C, 1 min, 56°C, 1 min; 72°C, 1 min (35 cycles) (60 mol/lit)
		Nested	GATGTCTTTAGCCACCCTTGATGT GAGCATTGATGGGCTTTTGACTTGC	640 bp	94°C, 1 min, 56°C, 1 min; 72°C, 1 min (35 cycles) (60 mol/lit)

*In-house designed primers for this study; PCR-polymerase chain reaction.

**Table 2:** Representation of strains of *H. pylori* after nested PCR restriction analysis.

Biopsy specimens	Restriction pattern of partial amplified genes of *H. pylori* by different restriction enzymes					
	*hsp60* by *Hin*d III		*ureC* (*glmM*) by *Hin*d III		*fliI* by *Mnl* I	
	Before therapy	After therapy	Before therapy	After therapy	Before therapy	After therapy
NUDG11	A	A	A	A	A	A
NUDG13	A	A	A	A	A	A
PUDG18	A	A	A	A	A	AA
NUDG35	A	A	A	A	A	A
NUDG37	A	A	A	A	A	A
PUDG43	A	A	A	A	A	A
NUDG46	A	A	A	A	A	AB1
PUDG47	A	—	A	—	A	—
PUDG57	A	A	A	A	A	A
NUDG61	A	A	A	A	A	A
PUDG63	A	A	A	A	A	A
NUDG64	A	A	A	A	A	A
NUDG67	A	A	A	AX	A	A
PUDG73	A	A	A	A	A	AB2
NUDG80	A	A	A	A	A	AA
PUDG81	A	A	A	A	A	A
PUDG85	A	—	A	—	A	—
PUDG88	A	A	A	A	A	AC
NUDG95	A	A	A	A	A	A
NUDG96	A	A	A	A	A	A
PUDG101	A	A	A	A	A	A
PUDG104	A	A	A	A	A	A
NUDG109	A	A	A	A	A	AA
PUDG115	A	A	A	A	A	A
PUDG118	A	A	A	A	A	A

Fragment length of AX-303 and 579 bp; AA-486 and 154 bp; AB1-386, 154, 55, and 45 bp; AB2-386, 134, 55, 45, and 22 bp; and AC-431, 300, and 154 bp.
